# Size of lymph-node metastases in prostate cancer patients undergoing radical prostatectomy: implication for imaging and oncologic follow-up of 2705 lymph-node positive patients

**DOI:** 10.1007/s00345-023-04724-1

**Published:** 2024-01-20

**Authors:** Fabian Falkenbach, Mykyta Kachanov, Sami-Ramzi Leyh-Bannurah, Tobias Maurer, Sophie Knipper, Daniel Köhler, Markus Graefen, Guido Sauter, Lars Budäus

**Affiliations:** 1 Martini-Klinik Prostate Cancer Center, University Hospital Hamburg-Eppendorf, Martinistrasse 52, 20246 Hamburg, Germany; 2 Institute of Human Genetics, University Medical Center Hamburg-Eppendorf, Hamburg, Germany; 3 Prostate Center Northwest, Department of Urology, Pediatric Urology and Uro-Oncology, St. Antonius-Hospital, Gronau, Germany; 4 Department of Urology, University Medical Center Hamburg-Eppendorf, Hamburg, Germany; 5 Department for Radiology and Nuclear Medicine, University Medical Center Hamburg-Eppendorf, Hamburg, Germany; 6 Department of Pathology, University Medical Center Hamburg-Eppendorf, Hamburg, Germany

**Keywords:** Pelvic lymph node dissection, Micrometastases, Prostate cancer, Radical prostatectomy, PSMA

## Abstract

**Background:**

Despite modern imaging modalities, lymph-node staging before radical prostatectomy (RP) remains challenging in patients with prostate cancer (PCa). The visibility of lymph-node metastases (LNMs) is critically influenced by their size.

**Objective:**

This study aims to describe the distribution of maximal tumor diameters (i.e., size) in LNMs of pN1-PCa at RP and its consequences on visibility in preoperative imaging and oncological outcomes.

**Design, setting, and participants:**

A total of 2705 consecutive patients with pN1-PCa at RP, harboring a cumulative 7510 LNMs, were analyzed. Descriptive and multivariable analyses addressed the risk of micrometastases (MM)-only disease and the visibility of LNMs. Kaplan–Meier curves and Cox analyses were used for biochemical recurrence-free survival (BCRFS) stratified for MM-only disease.

**Results:**

The median LNM size was 4.5mm (interquartile range (IQR): 2.0–9.0 mm). Of 7510 LNMs, 1966 (26%) were MM (≤ 2mm). On preoperative imaging, 526 patients (19%) showed suspicious findings (PSMA-PET/CT: 169/344, 49%). In multivariable analysis, prostate-specific antigen (PSA) (OR 0.98), age (OR 1.01), a Gleason score greater than 7 at biopsy (OR 0.73), percentage of positive cores at biopsy (OR 0.36), and neoadjuvant treatment (OR 0.51) emerged as independent predictors for less MM-only disease (p < 0.05). Patients with MM-only disease compared to those harboring larger LNMs had a longer BCRFS (median 60 versus 29 months, p < 0.0001).

**Conclusion:**

Overall, 26% of LNMs were MM (≤ 2mm). Adverse clinical parameters were inversely associated with MM at RP. Consequently, PSMA-PET/CT did not detect a substantial proportion of LNMs. LNM size and count are relevant for prognosis.

**Supplementary Information:**

The online version contains supplementary material available at 10.1007/s00345-023-04724-1.

## Introduction

Based on the limitations of conventional imaging [1, 2], clinical risk stratification before radical prostatectomy (RP) is performed by using risk calculators and nomograms [3, 4]. Higher specificity of lymph-node metastasis (LNM) imaging prior to RP (up to 90%), even in normal-sized lymph-nodes (LNs), can be achieved by PSMA-PET/CT. However, the limited sensitivity (down to 40%) restricts the benefit as an upfront staging procedure [5–8]. Reduced PSMA-tracer uptake due to lower prostate-specific antigen (PSA, < 10 ng/ml) and a lower Gleason score (< 8) [9] partly explain the limited sensitivity, especially in patients with a borderline indication for pelvic lymph-node dissection (PLND). In contrast, specificity and sensitivity for nodal metastases of 99% and 83%, respectively, were reported in the high-risk population included in the recent proPSMA-trial (although histological diagnosis confirmation was found only in 85/300 patients) [10]. In modern imaging, a minimum of 4.9 mm short-axis diameter of LNM is required for a detection rate of 90% in PSMA-PET/CT [11]. Consequently, EAU-guidelines conclude that LNMs with a short-axis diameter of under 5 mm may be missed by PSMA-PET/CT [12], and hence, new risk calculators incorporating PSMA-findings with improved area under the curve have been proposed [13]. However, the size distribution of LNMs at RP and its impact on diagnostic accuracy have not been described sufficiently so far. In detail, the prognostic implications of micrometastasis (MM, LNM ≤ 2 mm) and the chance of cure by RP with PLND alone for favorable nodal disease are unclear [14–16]. A previous analysis at our institution showed a gradual relationship between LNM size and biochemical recurrence-free survival (BCRFS) [17].

Therefore, we hypothesized that many LNMs at RP are small and cannot be detected by any upfront imaging. Therefore, this study assessed the distribution of LNM size and its diagnostic and prognostic implications in a large cohort of patients who underwent RP with PLND.

## Material and methods

### Patients

Between January 2014 and December 2021, 2705 consecutive patients harbored pN1-stage disease at RP with PLND. Nodal-negative patients were not included in this analysis. Six patients were excluded for false documentation or harboring LNMs of secondary malignancy. The indication for PLND was based on preoperative nomograms [3, 12]. For PLND, an extended bilateral pelvic template was dissected. Upfront imaging was performed in accordance with national and international guidelines [12, 18]. In our institution, cN1 does not automatically trigger neoadjuvant treatment. All patients provided informed consent for the procedures and data analyses.

### Outcomes of interest

First, this study describes the distribution of the maximal diameter (mm) of tumor deposits in lymph-nodes (hereinafter, “LNM size”) within pN1-patients at RP with PLND. Pre-/postoperative patient characteristics were prospectively collected within our institutional database (FileMaker Pro 10; FileMaker, Inc., Santa Clara, CA, USA). For precisely addressing tumor characteristics, the quantitative Gleason grading system (score) and the absolute amount of Gleason patterns 3, 4, and 5 at RP were included [19]. The pathological work-up of the LN material was previously described in 2014 and has not changed significantly since then [17]. In accordance with the common nomenclature for breast cancer [20], micrometastasis was defined as any LNM with a maximal diameter of 2 mm or less [21]. To account for multiple positive LN with different sizes in one patient, we calculated the biggest LNM in every patient. The term “micrometastases-only disease” (MM-only disease) described patients with one or more LNMs with a maximal tumor diameter of 2 mm or less.

Preoperative imaging addressing cN1 was recorded on a per-patient basis. If external imaging was available, a second reading by a dedicated uro-radiologist with more than 20 years of experience or an experienced nuclear medicine physician (for PET/CT) was performed prior to RP and was prospectively documented. The cN1-information of different imaging modalities was pooled.

For oncological outcomes, PSA recurrence was defined as a controlled postoperative serum (PSA ≥ 0.2 ng/ml) and adjuvant radiotherapy as radiation therapy administered within 180 days after RP. Adjuvant radiotherapy was performed with concomitant ADT as standard of care. Patients with PSA persistence after RP were included.

### Statistical analysis

The distribution of LNM size was graphically displayed, and the statistical significance of differences in medians and proportions was calculated using Kruskal–Wallis and Chi-squared tests. Kaplan–Meier plots graphically depict BCRFS with censoring at the time of loss to follow-up. The log-rank test was used for survival distribution, and Cox regression was used for BCRFS. Univariable and multivariable logistic regression models addressed the relationship between preoperative clinical risk factors and the prevalence of MM-only disease. For regression analysis, PSA, age, percentage of positive cores at biopsy, maximum LNM size (mm), LNM count, and cancer volume (ml) were coded continuously. Correspondingly, Gleason score at biopsy (Gleason score of ≤ 7 vs. > 7), neoadjuvant treatment (no vs. yes), D’Amico risk groups (low/intermediate vs. high), clinical stage (cT1 vs. cT2 vs. ≥ cT3), positive surgical margin at RP (no vs. yes), and adjuvant radiotherapy (no vs. yes) were coded categorically. The low- and intermediate-risk groups were pooled because of the low prevalence of low-risk diseases. All tests were two-sided, with a significance level set at p < 0.05.

For all statistical analyses, the R software environment for statistical computing and graphics (R version 4.1.2(2021-11-01), R Foundation for Statistical Computing) was used.

## Results

### Patients’ characteristics

Among the 2705 pN1-patients analyzed, a median of 19 LNs per patient (interquartile range (IQR): 14–27) and 2 LNMs per patient (IQR 1–3) were removed. A median of 16 LNs (IQR 11–22) per patient was analyzed by immunohistochemistry. Overall, 180,367 LNs and 7510 LNMs were analyzed. 1759 patients (65%) presented with high-risk disease and 916 (34%) with intermediate-risk disease prior to RP, according to D’Amico [22]. The median PSA and age at RP were 9.4 ng/ml (IQR 4.7–17.4 ng/ml) and 64.7 years (IQR 60–70 years) (Table 1), respectively. To improve surgical resectability or bridging time to RP, nearly one-quarter of the patients (724/2705, 27%) received neoadjuvant androgen deprivation therapy (ADT).

**Table 1:** Baseline characteristics of the patients (n = 2705)

Age at RP, years	Median, IQR	64.7 [60–70]
PSA at RP, ng/mL	Median, IQR	9.4 [4.7–17.4]
Risk group prior RP (D’Amico)		
Low	n, %	30 [1]
Intermediate	n, %	916 [34]
High	n, %	1759 [65]
pT Stage at RP (AJCC 2002)		
pT2	n, %	278 [10]
pT3a	n, %	668 [25]
pT3b	n, %	1702 [63]
pT4	n, %	57 [2]
Gleason score at RP		
≤ 6	n, %	1^#^ [0]
3 + 4	n, %	306 [11]
3 + 4 TG 5	n, %	237 [9]
4 + 3	n, %	298 [11]
4 + 3 TG 5	n, %	755 [28]
8	n, %	15 [1]
9–10	n, %	1093 [40]
LN count, n per patient	Median, IQR	19 [14–27]
Low-risk*	Median, IQR	19 [13–28]
Intermediate-risk*	Median, IQR	20 [14–27]
High-risk*	Median, IQR	23 [17–31]
LNM count, n per patient	Median, IQR	2 [1–3]
1	n, %	1313 [49]
2	n, %	491 [18]
3	n, %	324 [12]
≥ 4	n, %	577 [21]
Surgical margins		
Negative	n, %	1198 [44]
Positive	n, %	1500 [55]
Rx	n, %	7 [0]
Lymph-vessel invasion	n, %	1513 [56]
Neoadjuvant treatment	n, %	724 [27]
Kind of surgery		
Open RP	n, %	1799 [67]
Robot-assisted RP	n, %	906 [33]
Follow-up, months	median, IQR	37 [14 -61]
Patients with follow-up^†^	n, %	2454 [91]
Patients with adjuvant therapy	n, %	1046 [39]

*AJCC* American Joint Committee on Cancer, *PSA* prostate-specific antigen, *RP* radical prostatectomy, *TG *tertiary (Gleason) grade, *LN* lymph node, *LNM* lymph node metastasis

*Risk group according D’Amico prior RP

^#^The only patient with Gleason 6 disease underwent surgery after prior local therapy of the prostate

^†^Follow-up is defined at least one responded questionnaire after discharge. Adjuvant therapy was defined as adjuvant radiotherapy with concomitant ADT

### Size

**Fig. 1:**
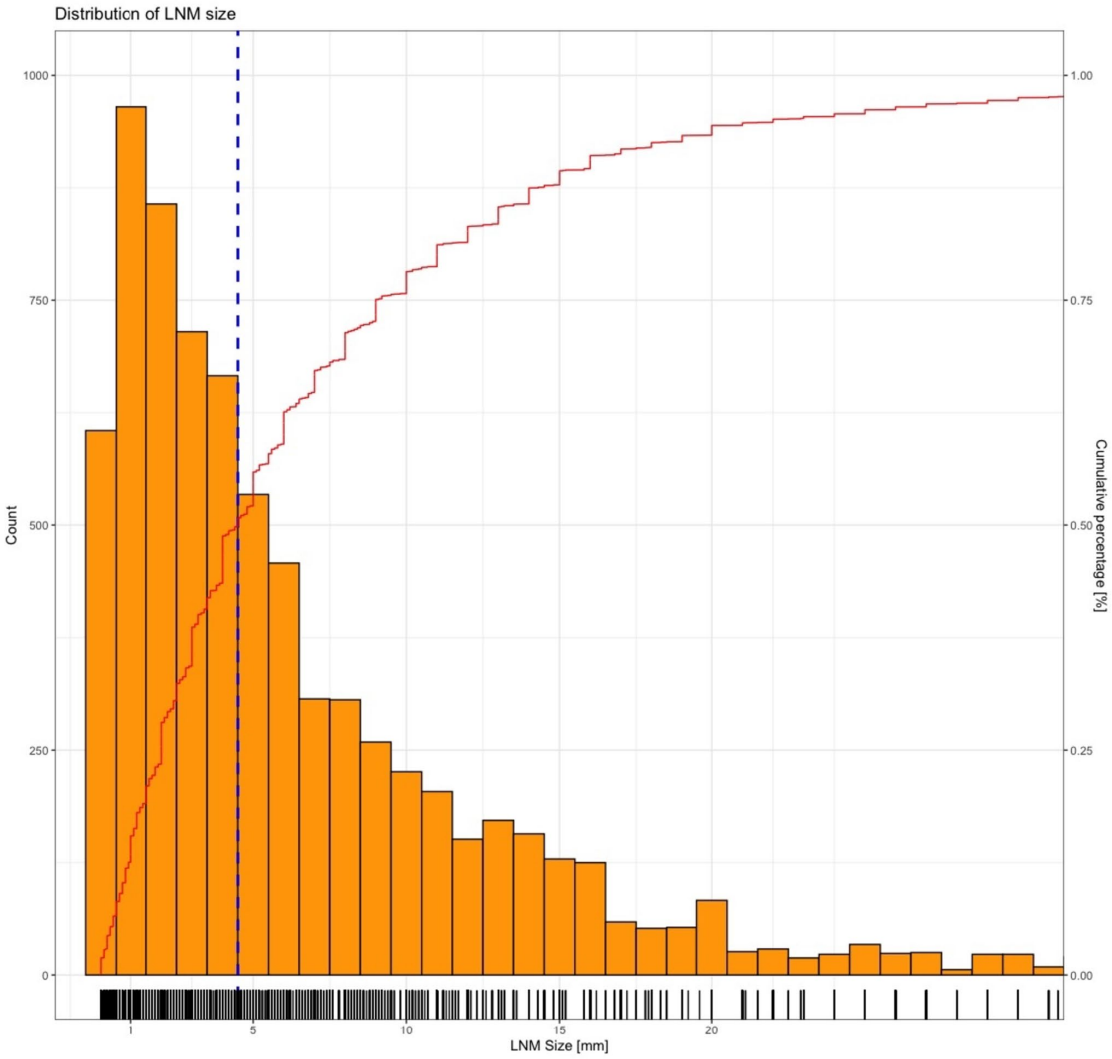
Histogram and rug plot of LNM count according to size (individual and cumulative size distribution, n = 2705). Blue line = median size of LNM; red line = cumulative percentage of LNM with the size or smaller (i.e., 75% of all LNM are 10 mm or smaller). The count (orange bars) is reported in absolute numbers and the cumulative percentage (red line) as a percentage (%)

The median LNM size was 4.5 mm (IQR 2.0–9.0 mm). Tabulating the distribution of LNM size according to incidence reveals a maximum of 2–3 mm (Fig. 1). Of 7510 LNMs, 1966 (26%) were MM (≤ 2 mm) and 4055 (54%) were smaller than 5 mm. Only 1639 of 7510 LNMs (22%) had a diameter > 10 mm. Interestingly, stratified by pT2 stage, PSA < 10 ng/ml, and Gleason score < 8, at least one MM was recorded in 200/278 (72%), 826/1424 (58%), and 973/1597 (61%) patients, respectively. In multivariable analysis, PSA (OR 0.98, CI 0.98–0.99), age at RP (OR 1.01, CI 1.00–1.03), Gleason score > 7 at biopsy (OR 0.73, CI 0.61–0.86), percentage of positive cores at biopsy (OR 0.36, CI 0.26–0.48), and neoadjuvant treatment (OR 0.51, CI 0.41–0.63) emerged as independent predictors for less MM-only pN1-disease (p < 0.05) on the basis of preoperative information. Accordingly, high-risk disease prior to RP based on the D’Amico groups was associated with less MM-only disease (OR 0.59, CI 0.40–0.56, p < 0.001). In addition, a palpable tumor before surgery predicted less MM-only disease (OR 0.69, CI 0.69–0.97, p < 0.05). Supplementary Table 2 provides a tabular overview of this regression model.

### Visibility

On preoperative imaging, 526 of 2705 patients (19%) showed suspicious findings (cN1). In total, 344 men were staged by PSMA-PET/CT, and of these, 169 men (49%) showed cN1. The overall detection rate of cN1 stratified for individual LNMs was size-dependent. For instance, in the cohort of LNM of 1 mm, cN1 was diagnosed in 5% (27/552), while imaging in the cohort of LNM of > 20 mm was positive in 67% (74/109) of patients prior to RP. In subgroup analysis for MM-only disease, PSMA-PET/CT was able to detect cN1 in 27% (20/74) of patients. In the cohort with PSA ≤ 10 ng/ml and low Gleason score < 8, PSMA-PET/CT had a mediocre detection rate for cN1-disease in 10 of 25 patients (40%; median LNM size here: 3.5 mm, IQR 2.0–4.8 mm).

### Oncological outcomes

**Fig. 2:**
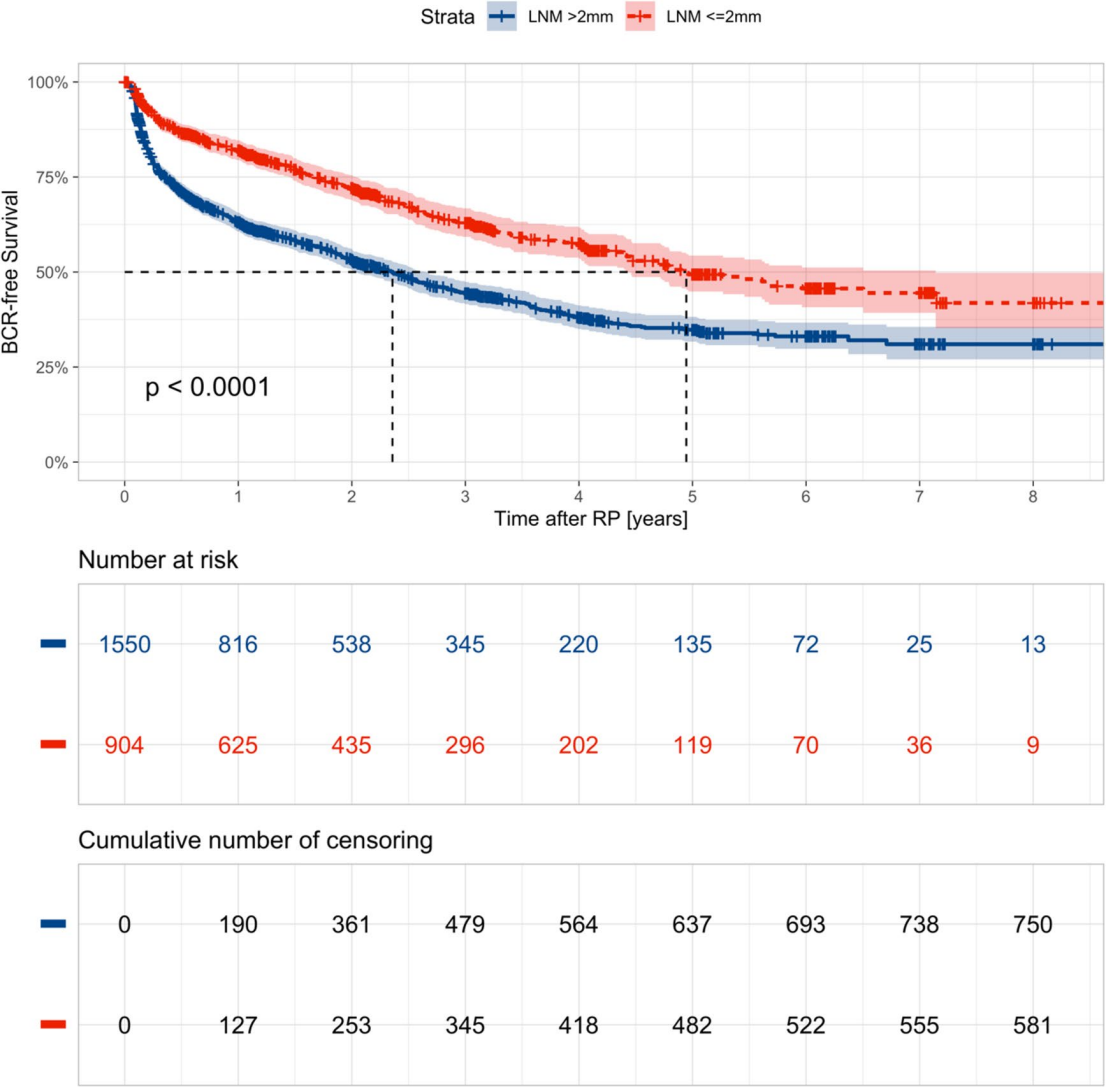
Kaplan–Meier analyses depicting biochemical recurrence–free survival rates in 2454 patients (all patients with follow-up) treated with RP, subdivided by patients with micrometastases-only (LNMs ≤ 2 mm) versus patients with at least one LNM > 2 mm

Follow-up data such as biochemical recurrence or death were available for 2454/2705 patients (91%). The median follow-up period was 37 months (IQR 14–61 months). The median BCRFS among all included patients was 39 months (IQR 35–43 months). A gradual decrease of BCRFS was observed with rising LNM size and count (Supplementary Figure 3). A significant BCRFS benefit for MM-only disease was observed (Fig. 2). In detail, the median BCRFS was 60 months for MM-only disease versus 29 months for patients with LNM > 2 mm (p < 0.0001). In multivariable Cox regression analysis, LNM size (HR 1.01 per mm, CI 1.00–1.02) and count (HR 1.03 per LNM, CI 1.01–1.05), PSA at RP (HR 1.00, CI 1.00–1.01), and local advanced disease (HR for pT3: 1.51, CI 1.19–1.92) were significant predictors of longer BCRFS (p < 0.05). In contrast, age at RP, neoadjuvant treatment, adjuvant radiotherapy, and bilateral-pN1 were not significant predictors. Supplementary Table 3 provides a tabular overview of this regression model. However, the proportional hazard function assumption could not be verified for all variates, i.e., LNM count, which limits the used Cox regression model.

## Discussion

The diagnostic accuracy of a given test is often reported based on its detection rates, sensitivity, and specificity. However, the pre-test probability, expressed by LNM incidence and size, is crucial to reporting detection rates. Therefore, the distribution of LNM sizes in a cohort provides the benchmark criteria for the true diagnostic accuracy of a test.

Specifically, the LNM size at RP is often small (median 4.5 mm), and in one-quarter of patients, there are MMs (≤ 2 mm, 26%). Jilg et al. reported a minimum of 4.9 mm for a detection rate of 90% by PSMA-PET/CT [11]. Similarly, our initial experiences in PSMA-PET/CT revealed median sizes of detected versus undetected LNMs of 13.6 mm and 4.3 mm, respectively [23]. Accordingly, the size distribution indirectly confirmed the findings of studies comparing LN staging using PSMA-PET/CT versus PLND. For example, in this analysis, 54% of LNMs (4055/7510) had a maximum tumor diameter of 5 mm. Therefore, based on the 4.9 mm threshold for PSMA-PET/CT, approximately half of LNMs in an all-comer population may be detected by upfront PSMA-PET/CT. Additionally, accounting for smaller LNMs that are detected by upfront PSMA-PET/CT at much lower rates, it might be expected that detection rates of roughly above 50% would be achieved. Interestingly, Esen et al. reported a detection rate of 53% in a recent retrospective series [24]. Similarly, two meta-analyses by Stabile et al. [25] and Tu et al. [26] established similar sensitivity rates for LNM of 58% and 63%, respectively.

In addition to size, other factors also influence the visibility of LNM. For example, recent PSMA-PET/CT data confirmed the relationship between higher pT-stage and higher Gleason score/cancer volume and visibility [9, 11]. Subsequently, a high proportion of high-risk patients, in whom larger LNM are more common, automatically improves the accuracy of reported imaging studies. Therefore, one strength of this study is the inclusion of all pN1-patients (35% low-/intermediate-risk) in a large consecutive cohort. This allowed for the assessment of the diagnostic performance of modern imaging modalities. As demonstrated in this real-world data, surgical staging by PLND remains the gold standard [12].

Within all pN1-patients, LNM size and count correlated with a shorter BCRFS in general and in MM-only disease in particular. This confirms a rather gradual rather than dichotomic progression of pN1-disease, as previously shown by Wilzcak et al. [17]. While the prognostic implications of any nodal-positive disease are undisputed, it can be hypothesized that some patients with low nodal metastatic burden are those in whom the curative effect of PLND is most realistic, as the plateau of the Kaplan–Meier curve in this study and other studies suggest [15, 27]. Prospective studies such as the PREDICT trial (NCT04269512) are necessary to assess the oncological value of PLND.

Despite its strengths, the current analysis was not devoid of limitations. First, only patients with localized or limited metastatic disease were referred for surgery (M1 in 2% of patients). Second, this comparative analysis was limited to only pN1-disease, and a comparator cohort was missing. Therefore, comparisons, especially of the BCRFS, and predictive power were highly limited. Third, LNM size is largely dependent on the pathological work-up, and in-vivo (i.e., imaging) vs. ex-vivo (i.e., pathology) measurements could not be equated due to several reasons, such as dehydration of specimens. However, the study results were consistent with other series [13, 16, 23]. Fourth, cancer-deposits in the LN are often not homogenously distributed within the LN; therefore, measurements of size are prone to interobserver variability [21]. There was no second-review of the LNM size for this analysis, and the size of LNs in patients without cancer was not assessed. However, the continuity of results with prior analyses at our institution further suggests the high stability of pathological procedures and reporting. Fifth, due to the abovementioned limited value of conventional and other PET imaging in nodal staging [28], the cN1-vs.-pN1 analysis is only useful in men staged with PSMA-PET/CT. Further analysis with more men staged by PSMA-PET is, therefore, necessary.

To conclude, diligent surgery and pathological work-up will most likely remain the cornerstones of prognostic risk assessment despite advances in molecular imaging.

## Conclusion

A significant number of patients harbor small LNMs and MMs. Based on their size, these small LNMs are generally not well detected by imaging. Interestingly, those patients in whom the indication for PLND is discussed the most are the ones who benefit the least from (molecular) upfront imaging since they tend to have often smaller and therefore poorly visible LNMs. Patients with low LNM size and count have a better prognosis than other pN1-patients.

## Supplementary Information

Below is the link to the electronic supplementary material.

Supplementary file1 (DOCX 5663 KB)

## Data Availability

Not available.
