# Supplementary material for: Size of lymph-node metastases in prostate cancer patients undergoing radical prostatectomy: implication for imaging and oncologic follow-up of 2705 lymph-node positive patients
Source: World J Urol. 2024 Jan 20;42(1):38. doi: 10.1007/s00345-023-04724-1 (PMC10799788; doi:10.1007/s00345-023-04724-1)
Supplement: Supplementary file 1 — Supplementary file1 (DOCX 5663 KB) [file 345_2023_4724_MOESM1_ESM.docx]

Supplementary Material

|  | **Univariable log. regression model** | | | | **Multivariable log. Regression model** | | | |
| --- | --- | --- | --- | --- | --- | --- | --- | --- |
| **Variables** | OR | CI 2.5% | CI 97.5% | p-value | OR | CI 2.5% | CI 97.5% | p-value |
| PSA (cont.) | 0.99 | 0.98 | 0.99 | <0.001 | **0.98** | **0.98** | **0.99** | **<0.001** |
| Age (cont.) | 1.02 | 1.01 | 1.03 | <0.01 | **1.01** | **1.00** | **1.03** | **<0.05** |
| **Gleason at Bx** |  |  |  |  |  |  |  |  |
| GG ≤7 | *Ref.* |  |  |  |  |  |  |  |
| GG >7 | 0.58 | 0.50 | 0.68 | <0.001 | **0.73** | **0.61** | **0.86** | **<0.001** |
| % pos. cores (cont.) | 0.22 | 0.17 | 0.30 | <0.001 | **0.36** | **0.26** | **0.48** | **<0.001** |
| **Neoadjuvant Treatment** |  |  |  |  |  |  |  |  |
| No | *Ref.* |  |  |  |  |  |  |  |
| Yes | 0.46 | 0.38 | 0.55 | <0.001 | **0.51** | **0.41** | **0.63** | **<0.001** |
| **D´Amico** |  |  |  |  |  |  |  |  |
| Low/ Intermediate* | *Ref.* |  |  |  |  |  |  |  |
| High | **0.47** | **0.40** | **0.56** | **<0.001** |  |  |  |  |
| **Clinical Stage** |  |  |  |  |  |  |  |  |
| cT1 | *Ref.* |  |  |  |  |  |  |  |
| cT2 | 0.76 | 0.64 | 0.89 | <0.001 | **0.82** | **0.69** | **0.97** | **<0.05** |
| ≥ cT3 | 0.44 | 0.30 | 0.62 | <0.001 | 0.79 | 0.54 | 1.15 | 0.23 |

Table 2: Uni- and multivariable logistic regression models predicting MM-only disease within pN1-patients with information prior RP / imaging (n=2705)

*OR= Odds ratio. CI= confidence interval. Ref. = Reference. RP = radical prostatectomy, PSA = prostate-specific antigen. *The risk groups of low / intermediate were pooled because of the low prevalence of low-risk disease. Furthermore, risk groups were excluded for multi-regression analysis presented here to avoid double inclusion of PSA, cT-Stage and Gleason group. However, in a separate multivariable logistic regression model with age, neoadjuvant treatment and percentage of positive cores, high-risk group remained a significant predictor (OR 0.59, CI 2.5%: 0.49, CI 97.5%: 0.69; p<0.001).*

|  | **Univariable Cox regression** | | | | **Multivariable Cox regression** | | | |
| --- | --- | --- | --- | --- | --- | --- | --- | --- |
| **Variables** | HR | CI 2.5 | CI 97.5 | p-value | HR | CI 2.5 | CI 97.5 | p-value |
| Max. LNM size, mm (cont.) | 1.03 | 1.02 | 1.03 | <0.001 | **1.01** | **1.00** | **1.02** | **<0.05** |
| LNM count (cont) | 1.07 | 1.05 | 1.08 | <0.001 | **1.03** | **1.01** | **1.05** | **<0.01** |
| Bilateral LNM | 1.36 | 1.2 | 1.54 | <0.001 | 0.97 | 0.84 | 1.13 | 0.69 |
| PSA at RP (*cont.)* | 1.01 | 1.00 | 1.01 | <0.001 | **1.00** | **1.00** | **1.01** | **<0.001** |
| Age at RP *(cont.)* | 0.99 | 0.99 | 1.00 | 0.14 |  |  |  |  |
| *Cancer Volume at RP, ml (cont.)* |  |  |  |  |  |  |  |  |
| Gleason Pattern 3 | 0.97 | 0.96 | 0.99 | <0.001 | **0.96** | **0.94** | **0.98** | **<0.001** |
| Gleason Pattern 4 | 1.03 | 1.02 | 1.03 | <0.001 | **1.02** | **1.01** | **1.03** | **<0.001** |
| Gleason Pattern 5 | 1.02 | 1.01 | 1.02 | <0.001 | 1.00 | 1.00 | 1.01 | 0.39 |
| pT Stage (AJCC) |  |  |  |  |  |  |  |  |
| pT2 | *Ref.* |  |  |  |  |  |  |  |
| pT3 | 1.87 | 1.48 | 2.36 | <0.001 | **1.51** | **1.19** | **1.92** | **<0.001** |
| pT4 | 2.54 | 1.66 | 3.89 | <0.001 | 1.43 | 0.9 | 2.27 | 0.13^#^ |
| PSM | 1.12 | 1 | 1.27 | 0.06 |  |  |  |  |
| Neoadjuvant Treatment | 1.09 | 0.95 | 1.24 | 0.23 |  |  |  |  |
| Adj. Radiotherapy* | 1.08 | 0.95 | 1.21 | 0.24 |  |  |  |  |

Table 3: Uni- and multivariable Cox regression models predicting biochemical recurrence-free survival after RP (n=2454, all patients with follow-up)

*HR = Hazard ratio. CI = confidence interval. Ref. = Reference. RP = radical prostatectomy. PSA = prostate-specific antigen. LN = lymph-node. LNM = lymph node metastasis. PSM = positive surgical margin. * Defined as radiation therapy within 180 days after RP. # Due to limited number of patients with pT4 (51 patients with pT4 and follow-up).*


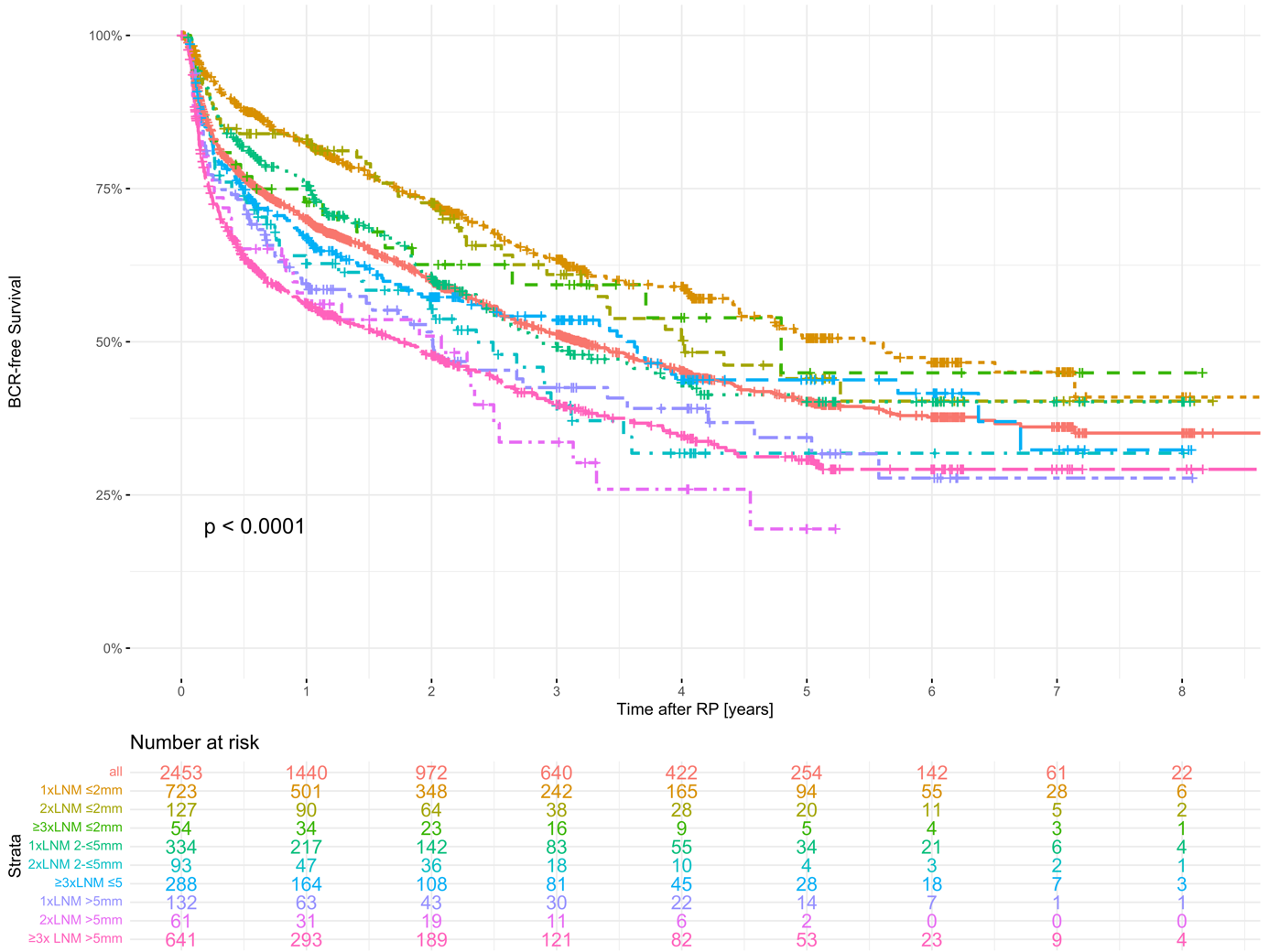


Figure 3: Kaplan-Meier curves depicting biochemical recurrence–free survival rates in 2454 patients (all patients with follow-up) treated with RP, subdivided by LNM count and maximal LNM size*.

** In case of more than one LNM, at least one LNM had the size of the sub-group and all subsequent LNM were the same size group or below. I.e. in the group “2x LNM 2-≤5mm” at least one LNM was between 2 to 5mm and the other LNM was 5mm or below. For illustrative purposes, a separate Kaplan-Meier curve depicticing MM-only vs. all other pN1 disease is found in the supplementary material. One patient without any size measurement was excluded from this analysis.*
